# Long-term impact of evidence-based quality improvement for facilitating medical home implementation on primary care health professional morale

**DOI:** 10.1186/s12875-018-0824-4

**Published:** 2018-08-31

**Authors:** Lisa S. Meredith, Benjamin Batorsky, Matthew Cefalu, Jill E. Darling, Susan E. Stockdale, Elizabeth M. Yano, Lisa V. Rubenstein

**Affiliations:** 1 RAND Corporation, 1776 Main Street, Santa Monica, CA 90407-2138 USA; 2 VA HSR&D Center for the Study of Healthcare Innovation, Implementation, and Policy, Los Angeles, CA USA; 3 TriveHive, Boston, MA USA; 4 USC Center for Economic and Social Research, Los Angeles, CA USA; 5 Department of Psychiatry and Biobehavioral Medicine, UCLA School of Medicine, Los Angeles, CA USA; 6 Department of Health Policy and Management, UCLA Fielding School of Public Health, Los Angeles, CA USA; 7 UCLA Schools of Medicine and Public Health, Los Angeles, CA USA

**Keywords:** Implementation, Evidence-based quality improvement, Patient-centered medical home, Primary care, Veterans

## Abstract

**Background:**

Poor morale among primary care providers (PCPs) and staff can undermine the success of patient-centered care models such as the patient-centered medical home that rely on highly coordinated inter-professional care teams. Medical home literature hypothesizes that participation in quality improvement can ease medical home transformation. No studies, however, have assessed the impact of quality improvement participation on morale (e.g., burnout or dissatisfaction) during transformation. The objective of this study is to examine whether primary care practices participating in evidence-based quality improvement (EBQI) during medical home transformation reduced burnout and increased satisfaction over time compared to non-participating practices.

**Methods:**

We used a longitudinal quasi-experimental design to examine the impact of EBQI (vs. no EBQI), a multi-level, interdisciplinary approach for engaging frontline primary care practices in developing evidence-based improvement innovations and tools for spread on PCP and staff morale following the 2010 national implementation of the medical home model in the Veterans Health Administration. The sample included 356 primary care employees (107 primary care providers and 249 staff) from 23 primary care practices (6 intervention and 17 comparison) within one Veterans Health Administration region. Three intervention practices began EBQI in 2011 (early) and three more began EBQI in 2012 (late). Three waves of surveys were administered across 42 months beginning in November 2011 and ending in January 2016 approximately 2 years 18 months apart. We used repeated measures analysis of the survey data on medical home teams. Main outcome measures were the emotional exhaustion subscale from the Maslach Burnout Inventory, and job satisfaction.

**Results:**

Six of 26 approved EBQI innovations directly addressed provider and staff morale; all 26 addressed medical home implementation challenges. Survey rates were 63% for baseline and 48% for both follow-up waves. Age was associated with lower burnout among PCPs (*p* = .039) and male PCPs had higher satisfaction (*p* = .037). Controlling for practice and PCP/staff characteristics, burnout increased by 5 points for PCPs in comparison practices (*p* = .024) and decreased by 1.4 points for early and 6.8 points (p = .039) for the late EBQI practices.

**Conclusions:**

Engaging PCPs and staff in EBQI reduced burnout over time during medical home transformation.

**Electronic supplementary material:**

The online version of this article (10.1186/s12875-018-0824-4) contains supplementary material, which is available to authorized users.

## Background

Delivering patient-centered accountable care across enrolled populations requires high functioning primary care practice teams as the basis for prevention, chronic disease care, and links to specialty, hospital, and long-term care [1]. Burnout among primary care providers (PCPs) (defined here as internal medicine and family physicians, physician assistants, or nurse practitioners) and their team members (often referenced as staff) can impede achievement of high functioning primary care [2, 3]. High levels of burnout in clinical teams are associated with poorer quality healthcare and decreased patient safety [4, 5]. In the work presented here, we test an approach, termed evidence-based quality improvement (EBQI), for accelerating change and maintaining morale in six engaged primary care sites during large scale implementation of a new team-based, patient centered primary care model (the patient centered medical home).

Burnout is a complex multi-dimensional condition characterized by emotional exhaustion (EE, the sense of being overwhelmed and exhausted), cynicism (feeling depersonalized and detached from the job), and professional efficacy (the lack of a sense of personal accomplishment related to work goals) [6, 7]. EE is considered to be the most central of the three components, is the most widely reported, and in some studies is the first domain that manifests when the full burnout syndrome is developing [8]. Burnout, and its EE component in particular, can be associated with lowered job satisfaction and increased job turnover [9, 10]. We assess EBQI outcomes based on PCP and staff EE and job satisfaction; we refer to these two concepts as indicators of PCP and staff morale.

Patient-centered medical home models can improve both patient outcomes [11] and provider morale [12, 13]. The model enhances the capabilities of primary care practices by linking all patients to both a continuity PCP and the PCP’s team. In the model, the patients work closely with their assigned PCP and team staff including e.g., a registered nurse, a health technician or licensed practical nurse, and a clerk. They also have access to extended team members that serve several PCP teams, such as a social worker, dietitian, and pharmacist. The model challenges traditional primary care disciplinary roles substantially by depending on strong team integration and functioning, with each member working “at the top of his or her license” [10, 11, 14].

Despite the enthusiasm for the medical home model, the stress inherent in transforming into high functioning, accountable primary care teams [10] runs the risk of increasing provider and staff burnout [9, 10] and reducing morale, thus impeding successful model implementation. The stresses are due not only to new working relationships, but to the many administrative challenges of adapting or replacing administrative systems such as scheduling, information systems, or performance monitoring. Yet it is particularly critical to maintain morale during transformation; falling levels can result in a vicious cycle of higher turnover within a primary care practice, less care continuity for patients, and greater burdens on continuing providers and staff. Just when expertise is most needed, trained team members may be replaced by less experienced professionals, causing continued turnover due to a poor work environment [6].

While much literature has examined the prevalence of burnout [15, 16] and its potential causes, very little has been written about how healthcare organizations might work to reduce it [9, 17]. Engagement in quality improvement has the potential to ease transformation by supporting development of local innovations for addressing transformation problems and for achieving needed care redesigns. Engagement might also empower teams to problem-solve in general, thus reducing feelings of stress, helplessness, and apathy [3] that can lead to burnout. Job satisfaction, in turn, is typically reduced when burnout occurs [18].

The Veterans Health Administration implemented its medical home model (termed Patient Aligned Care Teams, or PACT) nationally beginning in 2010 across its over 900 primary care sites [19, 20]. In addition to continuity team care, the implementation emphasized visit modalities other than face-to-face care, advanced or “open access” appointment scheduling, and new electronic performance measures accessible to sites on dashboards [21]. By engaging practices transforming into the new model in EBQI, a method tested both inside and outside the Veterans Health Administration [22], we aimed both to promote development of a high morale primary care quality improvement culture and to support systems re-engineering during transformation [22–30]. We previously documented high adherence to the EBQI model among the engaged sites [24]. We also found enhanced adoption of non-face-to-face care in EBQI compared to comparison sites [28]. We know of no prior work examining the impact of EBQI or similar approaches on morale.

EBQI aims to engage front-line clinical teams in developing innovations that reflect interdisciplinary input and are aligned with multi-level healthcare system leadership priorities. In this study, EBQI-engaged primary care sites developed quality councils [25] and participated in workgroups that generated proposals for innovations directed at medical home implementation; innovations are reviewed by regional leaders. Innovation teams discuss their approved projects in across-site telephone meetings and during yearly in person conferences that also engage regional leaders.

We addressed two questions: (1) Was transformation to the patient-centered medical home model associated with improved primary care practice morale (measured as emotional exhaustion and job satisfaction) over time? (2) Did engagement in EBQI improve morale among primary care practice’s providers and staff?

## Methods

### Design and setting

We compared changes over time in primary care provider and staff morale in EBQI-engaged practices and non-EBQI-engaged practices within the desert Pacific administrative region of the Veterans Health Administration. The desert pacific region breaks into five distinct healthcare networks, each including a medical center and community-based outpatient clinics. Three of the five networks agreed to participate and each selected a specific clinic in which to employ a Veterans Administration Improvement Laboratory facilitated EBQI approach. Three distinct primary care practices implemented EBQI-PACT beginning shortly after national PACT implementation in August 2010. Three additional primary care practices from the same three medical center-based networks initiated EBQI 19 months later (May 2012), for a total of six intervention practices. The 17 remaining comparison practices in the region underwent PACT implementation without EBQI.

### Exposure

EBQI promotes cross-discipline, data-driven problem solving in local primary care practices. EBQI aligns these local practices with organizational priorities to sustain successful QI innovations over time and spread them across teams and clinics. Specifically, the EBQI intervention focused on engaging and empowering front-line primary care teams with multi-level, interdisciplinary stakeholders in structured EBQI, and facilitated provider and staff initiated innovation projects. For EBQI practices, we engaged regional and local health system leaders and two frontline primary care practices from each of three of five local medical center-based Veterans Health Administration healthcare systems in the region.

The EBQI intervention included a proposal review and approval process that solicited brief innovation proposals from front-line providers and staff and provided approved innovation projects with additional support. Innovations could be proposed through either the EBQI practice’s quality council (supported by a quality council coordinator) [25] or through an across-EBQI site workgroup. The three medical center based networks supported the approved innovations with limited release time for the leaders of approved innovation projects, based on a prior Memorandum of Understanding initiated through the improvement laboratory with support of regional leaders. Regional leaders (administrative, quality, medical care, information technology, patient advocacy, pharmacy experts) served to set QI priorities by reviewing and rating the submitted proposals (a total of 71 during the time period reported here). We also convened yearly collaborative learning sessions across EBQI practices. We provided quality councils with local primary care site audit and feedback [25] comprised of practice level data on their patients, providers and staff, including provider and staff burnout, and assisted them in learning to access practice administrative data themselves.

Approved proposals (a total of 26) received a responsive innovation evidence review [31, 32] a budget based on the proposal budget request (average $12,000), and QI facilitation for project management and measures. Successful projects generated tools; if the innovation showed spread to at least one other site, the improvement laboratory assisted in formatting the tool and posting it on a Veterans Health Administration accessible SharePoint site (a total of 12 tools). An example of a tool is a step-by-step guide for enrolling and authenticating Veteran patients to use the online health portal for Veterans Health Administration. Additional volunteer projects could be undertaken by practices as well. All innovations addressed specific PACT-based problems or challenges. For example, one project addressed reducing homelessness among Veterans and another aimed to reduce unscheduled visits. There were six PACT team member-initiated, quality council approved projects designed to address provider and staff burnout and six volunteer projects addressing burnout completed during the reported time period.

### Participants

Our survey sample included all PACT PCPs (physicians, nurse practitioners, and physician assistants) and core PACT team staff (nurses, care/case managers, health educators, health technicians, medical assistants), as well as axillary staff such as dietitians/nutritionists, integrated mental health professionals, social workers, and pharmacists in EBQI and comparison sites, identified based on Veterans Health Administration’s electronic Primary Care Management Module. We excluded trainees from all disciplines.

### Data collection

We developed two versions of a survey: one for PCPs (Additional file 1) and one for staff (Additional file 2) that were identical in content where relevant. At each of three survey waves, we invited all PCPs and staff to complete the surveys online or to request a mail version. Surveys were administered from November 30, 2011 to March 30 2012 (wave 1), August 1, 2013 to January 15, 2014 (wave 2), and September 10, 2015 to January 8, 2016 (wave 3). We informed potential participants in an initial email request for participation that included consent language which made it clear that clicking the button to start the survey indicated that they have consented. All individuals who visited the web site or requested a mail version were entered into a drawing to win one of two iPad Air 2 s (in each wave).

### Measures

We examined two outcome measures. Emotional exhaustion burnout was the primary outcome, assessed with the 9-item subscale of the original Maslach Burnout Inventory (α = .92) [33, 34]. We scored the subscale by summing across the items rated on a 7-point (0–6) frequency scale (never, a few times a year, every month, a few times a month, every week, a few times a week, every day). Because of the overall burden of the study survey, we were unable to include items for the other two subscales (cynicism and professional efficacy). We measured past month job satisfaction with a single item, “Overall, I am satisfied with my job,” rated on a 5-point Likert scale.

We specified our 3-category *independent variable* using two binary variables to indicate each of the two EBQI intervention groups (early and late implementation) with the comparison group as the omitted category.

### Covariates

We controlled for age in years, gender with a binary indicator for male (vs. female), race/ethnicity (with binary indicators Latino and non-white/non-Latino relative to white as the omitted category), and number of years at the study clinic.

### Analysis

For the analysis, we included PCPs/staff who completed at least two of the three waves of surveys administered at baseline, approximately 20 and approximately 42 months later. We used three-wave repeated measures analyses in the form of a linear mixed model to estimate the total effect of EBQI vs. the comparison practice providers and staff on the emotional exhaustion subscale of burnout and the single item measure of job satisfaction [35]. We included main effects for survey wave and intervention group, their interaction, and random effects to account for the repeated measures within individual and the clustering of individuals within clinics controlling for covariates.

## Results

### Survey and sample characteristics

The overall response rates were 63% for baseline, and 48% for both follow-up waves. Response rates for professionals in EBQI practices were the same as professionals in comparison practices at waves 1 and 2 but were higher in wave 3 (38% vs. 55%). The analysis sample included 356 professionals (107 PCPs and 249 staff). Response rates were higher for staff compared with PCPs for all three waves. There were no significant differences between the groups of providers (Table 1) with the exception of years in the clinic; EBQI providers spent 8 years on average in their assigned practices compared with 5.2 years for providers in comparison practices (*p* = .011).

**Table 1:** Demographic and Professional Characteristics of Primary Care Employees by Study Group

Characteristic	EBQI (*n* = 181)^a^	Comparison (*n* = 175)^a^	Full Sample (*n* = 356)^a^
Female, no. (%)	124 (67)	121 (70)	245 (69)
Latino, no. (%)	20 (11)	15 (9)	35 (10)
Non-white non-Latino, n (%)	87 (47)	71 (41)	158 (44)
Age, mean (SD), y	47.4 (10.0)	47.6 (11.0)	46.8 (10.9)
Years in clinic, mean (SD)	8.0 (8.1)	5.2 (7.1)	7.0 (7.7)*
Job type, no. (%)			
Physician			75 (21)
General practice/family medicine	3 (2)	7 (4)	10 (3)
Internal medicine	39 (22)	20 (11)	59 (17)
Other specialty^a^	3 (2)	3 (2)	6 (2)
Nurse practitioner	12 (7)	16 (9)	28 (8)
Physician assistant	2 (1)	2 (1)	4 (1)
Registered nurse	48 (27)	49 (28)	97 (27)
Licensed practical/vocational nurse	37 (20)	41 (23)	78 (22)
Mental health professional	4 (2)	4 (2)	8 (2)
Social worker	1 (1)	5 (3)	6 (2)
Dietician or nutritionist	5 (3)	3 (2)	8 (2)
Pharmacist	11 (6)	12 (7)	23 (6)
Health/medical technician/assistant/clerk	8 (4)	2 (1)	10 (3)
Clerk	8 (4)	11 (6)	19 (5)

*p < .01, where EBQI and comparison employees differ significantly for these variables

^a^Other specialties include rheumatology, geriatrics, and infectious diseases

### Effect of EBQI-PACT on EE burnout over time

**Fig. 1:**
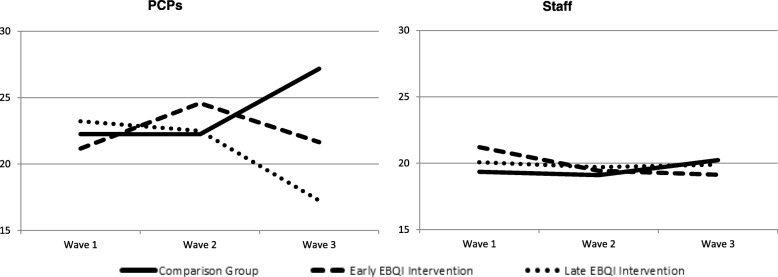
Change in Emotional Exhaustion Burnout Across Wave by Intervention Group for Primary Care Providers (PCPs) and Staff

Figure 1 illustrates the unadjusted findings for EE over time for each of the three groups (early EBQI, late EBQI, and comparison practices) separately for PCPs and staff. We found large intervention effects over time for PCP burnout, particularly by wave 3, but little or no change over time in staff EE.

Table 2 shows the estimated effect of EBQI and the changes over time based on difference in differences analyses in each of the three groups stratified by provider type after adjusting for covariates. From wave 1 to wave 3, relative to the comparison practices and accounting for each practices’ baseline EE score, the early EBQI-PACT practices had lower EE scores over time by 1.42 points (not significant) and the late implementation EBQI-PACT practices had significantly lower EE scores by 6.82 points (*p* = .039), a difference equivalent to one-half a standard deviation.

**Table 2:** Results from Regression Models for Change in Emotional Exhaustion Burnout and Job Satisfaction, Score (CI)

Variable	Emotional Exhaustion Burnout		Job Satisfaction	
	PCPs	Staff	PCPs	Staff
Intercept	40.38 (25.81, 54.94)	14.88 (7.34, 22.43)	2.38 (1.23, 3.54)	4.26 (3.72, 4.80)
Difference in Differences (Wave 3 – Wave 1)				
Early EBQI-PACT vs. Comparison	−1.42 (− 8.74, 5.90)	− 1.44 (−7.02, 4.15)	− 0.12 (− 0.77, 0.54)	0.36 (− 0.16, 0.88)
Late EBQI-PACT vs. Comparison	− 6.82 (− 13.29, − 0.35)*	− 1.30 (− 6.72, 4.11)	0.22 (− 0.38, 0.82)	0.08 (− 0.43, 0.60)
Change within Group (Wave 3 – Wave 1)				
Comparison Group	4.96 (0.66, 9.25)*	0.84 (−2.28, 3.96)	− 0.21 (− 0.61, 0.18)	−0.39 (− 0.68, − 0.10)**
Early EBQI-PACT Intervention	3.54 (− 2.53, 9.60)	− 0.60 (− 5.28, 4.08)	−0.33 (− 0.86, 0.20)	−0.03 (− 0.47, 0.40)
Late EBQI-PACT Intervention	− 1.86 (− 6.84, 3.11)	−0.46 (− 4.98, 4.06)	0.01 (− 0.45, 0.47)	−0.31 (− 0.74, 0.12)
Change from Wave 1 (Comparison Group)				
Wave 1 (Reference group)	–	–	–	–
Wave 2	1.75 (−2.18, 5.69)	0.03 (− 2.77, 2.83)	−0.09 (− 0.43, 0.26)	−0.22 (− 0.48, 0.05)
Wave 3	4.96 (0.66, 9.25)*	0.84 (− 2.28, 3.96)	− 0.21 (− 0.61, 0.18)	−0.39 (− 0.68, − 0.10)**
Covariates				
Age, y	− 0.29 (− 0.57, − 0.01)*	0.06 (− 0.08, 0.21)	0.02 (0.00, 0.04)	0.00 (− 0.01, 0.01)
Male	−3.71 (−8.30, 0.89)	−1.17 (− 4.78, 2.43)	0.39 (0.02, 0.75)*	0.08 (− 0.17, 0.33)
Latino	−5.50 (− 13.16, 2.15)	−2.25 (− 6.72, 2.22)	0.36 (− 0.26, 0.97)	0.00 (−0.33, 0.33)
Non-white, Non-Latino	0.64 (−4.38, 5.66)	2.79 (−0.41, 5.99)	−0.19 (− 0.59, 0.20)	−0.18 (− 0.40, 0.04)
Years at clinic	− 0.11 (− 0.37, 0.16)	0.11 (− 0.13, 0.36)	0.01 (− 0.01, 0.03)	0.00 (− 0.01, 0.02)

*p < .05; **p < .01

Table 2 also shows absolute change over time between wave 1 and wave 3 within each of the three groups after adjusting for covariates. PCPs in comparison practices had increased EE scores over time of 4.96 points (*p* = .024). This five-point increase is equivalent to 0.40 of a standard deviation on the 0–54 point EE scale. Though not significant, scores also increased for PCPs in early EBQI-PACT practices (by 3.54 points) but decreased by 1.86 points for late EBQI-PACT practices.

Table 2 further shows the absolute differences in EE between the comparison group and the early and late EBQI groups by survey wave, after adjusting for covariates (i.e., without taking account of baseline practice differences). There were no significant differences between the comparison practices and the early EBQI-PACT practices at any survey wave. However, the late EBQI-PACT practices showed a marginally significant difference in EE by wave 3 of 6.23 points lower than comparison practices (*p* = .073). Among the set of covariates, only age was significantly associated with EE over time for PCPs; older PCPs had lower EE scores by 0.29 points (*p* = .039).

### Effect of EBQI on job satisfaction over time

**Fig. 2:**
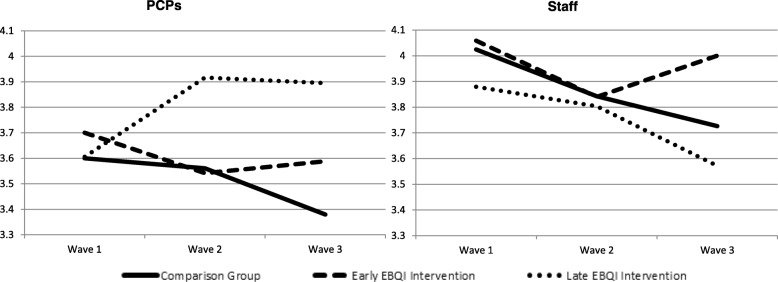
Change in Job Satisfaction Scores Across Wave by Intervention Group* for Primary Care Providers (PCPs) and Staff

The unadjusted patterns of effects for job satisfaction (Fig. 2), and adjusted estimates appear in Table 2. We found no significant differences in the difference in differences analyses, and no significant changes in PCP job satisfaction in EBQI versus comparison practices. In adjusted results testing absolute within group differences over time, we observed a significant decrease in satisfaction over time for staff in comparison practices and no change over time for staff in early EBQI practices or for change over time for staff in late EBQI practices. Specifically, staff in comparison practices had significantly reduced job satisfaction by 0.39 points (*p* = .008), but for those in early implementation of EBQI group, this effect was near zero, and in the late EBQI group, burnout decreased by 0.31 points though not significantly. Of the covariates, only gender for staff was significantly associated with satisfaction for PCPs; men had satisfaction scores 0.39 points higher (*p* = .037), approximately 35% of a standard deviation.

## Discussion

Experts agree that high functioning primary care teams, such as those featured in patient-centered medical home models, are essential for delivery of high value accountable care across enrolled patient populations. Yet achieving transformation to these models is challenging. We examined whether EBQI [24] was effective in reducing PCP/staff burnout [33, 34] or increasing satisfaction during national transformation to the Veterans Health Administration’s medical home model. We used a rigorous quasi-experimental design that compared change over time in intervention clinics (EBQI in the context of the medical home) vs. comparison clinics (medical home alone) within the first five years of transformation. We found a significant EBQI effect on reducing burnout among PCPs, but no significant effect for early EBQI-PACT practices or for late EBQI-PACT practices relative to comparison practices.

Our findings are important because burnout and dissatisfaction are associated with adverse consequences for patient care [21, 36] and with increased job turnover across industries [2, 15, 37, 38]. Worsened burnout and job satisfaction can become acute during transformation to the patient-centered medical home [2], threatening the success of the new model. Only PCPs had significantly reduced EE burnout but not staff. Conversely, job satisfaction was better over time only for staff but not for PCPs. A possible explanation for this finding is that because PCPs have overall responsibility for creating and leading functional teams, their role in PACT is inherently more stressful than for staff which makes them more prone to burnout. This longitudinal observational study is the first to test whether a systematic approach to engaging front-line primary care practices in inter-professional medical home-related quality improvement innovation might ameliorate or reduce burnout and increase job satisfaction during the difficult work of implementing the medical home model.

This study followed PCPs and staff for nearly five years after national patient-centered medical home implementation in Veterans Health Administration, far longer than in previous studies of change in burnout and satisfaction, and began over a year after medical home implementation was nationally mandated in Veterans Health Administration. The study thus spanned the initial five years of medical home transformation. Additionally, unlike most prior work, this study assessed PCPs and staff separately, given that medical home implementation role and task changes are substantially different for PCPs compared to staff [39, 40]. Further, we do not know if the innovation efforts were initiated by PCPs, other types of providers and staff, or with equal input across type of provider/staff. It is possible that the reason we found a positive impact on PCP burnout but not staff burnout is that because most of the innovations were spearheaded by PCPs, the psychological investment was greater for them. Greater psychological investment is likely associated with more engagement in change. Heavier investments, particularly by enthusiastic employees, have been found to have positive associations with a number of work characteristics including less role ambiguity and greater autonomy [41]. Although a few studies have found reductions in burnout over time post-medical home implementation, these have been limited to one or two years [11, 13]. Two years may be too short; one study found that practices that were relatively more successful at implementing the medical home model did so only after more than two years of implementation [42]. Adopting new models of care requires changing the culture of practice and getting past initial change fatigue as well as having an infrastructure that enables transformation [43]. Health systems should consider tracking provider engagement and well-being (including burnout levels) along with standard institutional measures such as cost, quality, and patient satisfaction) [17] following medical home implementation.

Major change takes time and resources, particularly at the organizational level. This is possibly why we did not observe a statistically significant difference between early EBQI and the comparison group for either EE or job satisfaction. National Veterans Health Administration PACT implementation, experienced by both intervention and comparison groups in this study, included a well-documented care model, mandated changes in staffing, time-limited increases in special funding for implementation, data resources including a national dashboard with primary care practice level PACT process measures, web-based tools, redesign collaboratives, and substantial PACT training opportunities for providers and staff. Nevertheless, without EBQI, burnout increased for comparison practices. EBQI appeared to have a protective effect, avoiding significant worsening of morale (burnout and satisfaction) among the early EBQI group and improving it in the late EBQI group.

In a recent meta-analysis of interventions to prevent and reduce physician burnout, West and colleagues [44] identified a total of 15 randomized trials and 37 cohort studies that were relevant. They found that interventions that were focused on the individual as well as interventions that were focused on structural changes or at the organizational-level were associated with clinically meaningful reductions in burnout. The statistical review of these studies is the first step toward understanding how to engage the healthcare workforce in strategies to boost morale and prevent or minimize burnout and improve morale under hyperkinetic circumstances. The EBQI approach used here includes both individual and organizational strategies (i.e., interdisciplinary leadership participation, management assistance from quality council coordinators, and technical assistance from improvement laboratory researchers) by engaging individual providers as well as administrators and leadership in the transformation process. The approach emphasizes psychological factors that can make adaptation to change a more positive experience [45, 46]. It also emphasizes using systematic, interdisciplinary, organizationally aligned redesign to solve the inevitable issues arising from transformation. The redesign efforts used local innovation, formal quality improvement methods, outcome measures, and tool-based spread. Our findings suggest that the EBQI approach holds promise for putting the joy back into practice [3], as well as for more rapid implementation of medical home features [28].

Our study has a number of important limitations warranting caution in interpreting results. First, our design was quasi-experimental; participating medical center-based network leaders chose EBQI practices for a variety of reasons. Our repeated measures analyses and covariates control for major differences between EBQI and comparison practices at baseline. However, our sample size limited our ability to control for all possible differences and effects of unmeasured variables cannot be ruled out. Our restricted sample size also prevented disaggregating the sample staff to examine specific experiences of mental health providers or pharmacists. Also, while our self-report burnout measure could be biased, we would expect this effect to influence baseline as well as follow-up results. Additionally, we measured burnout based on only the emotional exhaustion subscale of the Maslach burnout inventory; we did not administer the other two subscales at waves 2 and 3. Our previous analyses of wave 1 data indicated that the emotional exhaustion subscale was highly correlated with the other two Maslach subscales (cynicism and professional efficacy) and more sensitive to variations in PACT.

## Conclusion

Engaging PCPs and staff in EBQI reduced burnout over time during medical home transformation. Observed effects may have been due to more rapid solving of medical home-related problems and challenges through system redesign or the other types of quality improvement innovations undertaken by EBQI practices, including innovations directly aimed at addressing burnout. They may also have been due to increased engagement of PCPs and staff in medical home implementation. Healthcare systems should consider EBQI as a systematic method for assisting with large organizational changes, such as medical home implementation.

## Additional files

Additional file 1: PACT Clinician Survey. Survey instrument for primary care providers. (PDF 609 kb)

Additional file 2: PACT Staff Survey. Survey instrument for other staff that work with primary care. (PDF 574 kb)

## Abbreviations

EBQI: Evidence-based quality improvement
EE: Emotional exhaustion
medical home: PACT: Patient Aligned Care Teams; Patient-Centered Medical Home
PCP: Primary Care Providers
VA: Veterans Administration
VHA: Veterans Health Administration
