# Supplementary material for: Long-term impact of evidence-based quality improvement for facilitating medical home implementation on primary care health professional morale
Source: BMC Fam Pract. 2018 Aug 31;19:149. doi: 10.1186/s12875-018-0824-4 (PMC6119243; doi:10.1186/s12875-018-0824-4)
Supplement: Supplementary file 2 — PACT Staff Survey. Survey instrument for other staff that work with primary care. (PDF 574 kb) [file 12875_2018_824_MOESM2_ESM.pdf]

VAIL
Veterans Assessment and Improvement Laboratory
VISN 22

PACT
PATIENT ALIGNED CARE TEAM

VISN 22 Primary Care Clinician and Staff Survey

Staff Version

About this survey:
This survey asks about your experiences as a member of a primary care team. All primary care clinicians and other clinical staff members of primary care teams at facilities within VISN 22 are being invited to take part. We estimate that it will take about **20 minutes** to complete this survey, depending on your answers.

About your participation:
This survey is strictly voluntary; your participation decision will have no adverse affect on you professionally or personally. Answering survey questions will constitute agreement to participate. For more detailed information about the survey and your participation, please refer to the Question and Answer addendum.

Before you begin, please indicate below whether you would like us to enter you in our lottery for one of two iPad 2s. The winner will be chosen at random from among those who check "yes". Lottery drawings will occur on **December 16, 2011** and **March 9, 2012**. You do not need to participate in the survey to enter the lottery, and your participation decision will not affect your chances of winning. Survey participation is strictly separate from lottery participation.

☐ **Yes, please enter me in the lottery to win one of two iPad 2s**

☐ **No, I do not wish to be entered the lottery**

If you have questions about this survey, please contact Lisa Meredith, lead RAND investigator, at 310-393-0411 ext. 7365 or seidel@rand.org.

A. Professional Background and Clinic Characteristics

1. Are you a staff member in a VA primary care clinic?

   (Check One)
   - ☐ Yes *(Please continue)*
   - ☐ No
     - ↳ *(Thank you. We do not need you to fill out this survey, but appreciate your interest. You are still eligible for the iPad 2 lottery.)*

2. Which job title comes closest to your current job?

Job title	*(Circle One)*
Nurse Care/Case Manager	1
Registered Nurse (roles other than Care Manager)	2
Licensed Practical Nurse, Licensed Vocational Nurse, or Certified Nursing Assistant	3
Dietician or Nutritionist	4
Health Educator	5
Health Technician, Medical Assistant, or Medical Technician	6
Mental Health Professional	7
Social Worker (primary job is other than mental health counseling)	8
Pharmacist	9
Other (*Please specify: ____*)	10

3. How many years have you been employed at this VA clinic?

   WRITE IN # OF YEARS: _______

4. Are you currently a full-time or part-time employee at the VA?

   (Check One) ☐ Full-time ☐ Part-time

5. In a typical week, how many different primary care clinicians (e.g., physicians, physician assistants, or nurse practitioners) do you work with?

   WRITE IN # OF CLINICIANS: _______

B Team/Teamlet Composition and Characteristics

A PACT teamlet typically consists of a primary care provider (MD, NP, or PA), registered nurse, clinical associate (LPN or medical assistant/health technician), and administrative associate who are assigned to care for a defined patient group or panel in continuity.

1. Are you currently a member of a PACT teamlet? *(Please refer to teamlet description above.)*

(Check one) ☐ Yes* ☐ No** ☐ Not sure**

* If Yes, how many PACT teamlets are you a member of?

WRITE IN # OF TEAMLETS: _____

** If No or Not sure, are you aware of any plans for you to be included in a PACT teamlet?

(Check one) ☐ Yes ☐ No ☐ Not sure

If you are not a member of a teamlet, skip to Question 3 on the next page. Otherwise, please continue.

2. To what extent does <u>your teamlet</u> rely on you to help accomplish the following primary care activities? *(If you are a member of more than one teamlet, please answer with respect to the teamlet you spend the most time working with.)*

Primary care activities	Does not rely on me at all	Relies on me slightly	Relies on me somewhat	Relies on me a great deal
a. Gathering patient preventive services utilization history (e.g., immunization history)	☐	☐	☐	☐
b. Screening patients for diseases (e.g., doing a depression screen)	☐	☐	☐	☐
c. Evaluating patients and making treatment decisions	☐	☐	☐	☐
d. Intervening on patient lifestyle factors (e.g., diet, smoking cessation)	☐	☐	☐	☐
e. Educating patients about disease-specific self-care activities (e.g., foot care in diabetes)	☐	☐	☐	☐
f. Educating patients about medications	☐	☐	☐	☐
g. Responding to prescription refill requests	☐	☐	☐	☐
h. Receiving messages from patients (other than requests for prescriptions)	☐	☐	☐	☐
i. Resolving messages from patients (other than requests for prescriptions)	☐	☐	☐	☐
j. Handling forms for patients (e.g., disability documentation)	☐	☐	☐	☐
k. Tracking patient diagnostic data (e.g., labs, radiology studies)	☐	☐	☐	☐
l. Responding to patient diagnostic and treatment data (e.g., labs, radiology studies)	☐	☐	☐	☐
m. Following-up on referrals (e.g., to specialists)	☐	☐	☐	☐
n. Responding to requests for Home Health Care orders	☐	☐	☐	☐

3. Please identify which of the following health service professionals are members of your <u>primary care team</u>. *This refers to any colleagues whom you communicate with <u>during a typical month</u> to help provide primary care for patients.*

Potential primary care team members	(Check if a member of your team)
Physician	
Nurse Practitioner	
Physician Assistant	
Nurse Care/Case Manager	
Registered Nurse (roles other than Care Manager)	
Licensed Practical Nurse, Licensed Vocational Nurse, or other Nurse	
Health Technician, Medical Assistant, or Medical Technician	
Social Worker (primary job is other than mental health counseling)	
Mental Health Professional	
Pharmacist	
Dietician or Nutritionist	
Health Educator	
Resident/Trainee (Medicine, Nursing, or other)	
Medical subspecialist(s) (*Specify:* ______________________________)	
Medical subspecialist(s) (*Specify:* ______________________________)	
Other (*Specify:* ______________________________)	
Other (*Specify:* ______________________________)	

4. Considering the members of your primary care team whom you listed in the previous question, please indicate how much you agree or disagree with the following statements.

	Strongly Disagree	Disagree	Neither disagree nor agree	Agree	Strongly agree
a. Members of our team actively share their special knowledge and expertise with one another.	☐	☐	☐	☐	☐
b. Some members of this team lack the knowledge and skills that they need to do their parts of the team's work.	☐	☐	☐	☐	☐
c. Members of this team have more than enough talent and experience for the kind of work that we do.	☐	☐	☐	☐	☐
d. Our team is quite skilled at capturing the lessons that can be learned from our work experiences.	☐	☐	☐	☐	☐
e. How seriously a member's ideas are taken by others on our team often depends more on who the person is than on how much he or she actually knows.	☐	☐	☐	☐	☐
f. Everyone in this team has the special skills that are needed for team work.	☐	☐	☐	☐	☐

If you <u>are a member of a teamlet</u>, skip to <u>Question 1 on the next page</u>. Otherwise, please continue.

5. **To what extent does your <u>primary care team</u> rely on you to help accomplish the following primary care activities?** *(Once again, please consider the primary care team members you listed in a previous question.)*

Primary care activities	Team does not rely on me at all	Team relies on me slightly	Team relies on me somewhat	Team relies on me a great deal
a. Gathering patient preventive services utilization history (e.g., immunization history)	☐	☐	☐	☐
b. Screening patients for diseases (e.g., doing a depression screen)	☐	☐	☐	☐
c. Evaluating patients and making treatment decisions	☐	☐	☐	☐
d. Intervening on patient lifestyle factors (e.g., diet, smoking cessation)	☐	☐	☐	☐
e. Educating patients about disease-specific self-care activities (e.g., foot care in diabetes)	☐	☐	☐	☐
f. Educating patients about medications	☐	☐	☐	☐
g. Responding to prescription refill requests	☐	☐	☐	☐
h. Receiving messages from patients (other than requests for prescriptions)	☐	☐	☐	☐
i. Resolving messages from patients (other than requests for prescriptions)	☐	☐	☐	☐
j. Handling forms for patients (e.g., disability documentation)	☐	☐	☐	☐
k. Tracking patient diagnostic data (e.g., labs, radiology studies)	☐	☐	☐	☐
l. Responding to patient diagnostic and treatment data (e.g., labs, radiology studies)	☐	☐	☐	☐
m. Following-up on referrals (e.g., to specialists)	☐	☐	☐	☐
n. Responding to requests for Home Health Care orders	☐	☐	☐	☐

C. Individual Perceptions and Experiences

1. Please indicate how much you agree or disagree with the following statements as they apply to you over the last month:

	Strongly Disagree	Disagree	Neither disagree nor agree	Agree	Strongly agree
a. Overall, I am satisfied with my job.	☐	☐	☐	☐	☐

If you are not a member of a teamlet, skip to Question 2 on the next page. Otherwise, please continue.

	Strongly Disagree	Disagree	Neither disagree nor agree	Agree	Strongly agree
b. Overall, I am satisfied with how my teamlet members work together.	☐	☐	☐	☐	☐

2. Please indicate how frequently you experience each of the following feelings or attitudes.

	Never	A few times a year	Every month	A few times a month	Every week	A few times a week	Every day
a. I feel emotionally drained from my work.	☐	☐	☐	☐	☐	☐	☐
b. I feel exhilarated when I accomplish something at work.	☐	☐	☐	☐	☐	☐	☐
c. I doubt the significance of my work.	☐	☐	☐	☐	☐	☐	☐
d. I feel used up at the end of the workday.	☐	☐	☐	☐	☐	☐	☐
e. Working with people all day is really a strain for me.	☐	☐	☐	☐	☐	☐	☐
f. I feel burned out from my work.	☐	☐	☐	☐	☐	☐	☐
g. I feel fatigued when I get up in the morning and have to face another day on the job.	☐	☐	☐	☐	☐	☐	☐
h. I have accomplished many worthwhile things in this job.	☐	☐	☐	☐	☐	☐	☐
i. I feel frustrated by my job.	☐	☐	☐	☐	☐	☐	☐
j. I feel I'm working too hard on my job.	☐	☐	☐	☐	☐	☐	☐
k. Working with people directly puts too much stress on me.	☐	☐	☐	☐	☐	☐	☐
l. I feel like I'm at the end of my rope.	☐	☐	☐	☐	☐	☐	☐
m. In my opinion, I am good at my job.	☐	☐	☐	☐	☐	☐	☐
n. I have become less enthusiastic about my work.	☐	☐	☐	☐	☐	☐	☐
o. I just want to do my job and not be bothered.	☐	☐	☐	☐	☐	☐	☐

3. Please respond to the following statements about the implementation of PACT.

	Strongly disagree	Disagree	Neither disagree nor agree	Agree	Strongly agree
a. As we implement PACT, I feel I can handle my role with ease.	☐	☐	☐	☐	☐
b. There are some PACT-related tasks that I should be doing, but don't think I can do well.	☐	☐	☐	☐	☐
c. I have the skills that are needed to make my role in PACT successful.	☐	☐	☐	☐	☐
d. There are some tasks expected of my role in PACT that I don't have time for.	☐	☐	☐	☐	☐
e. My past experiences make me confident that I will be able to perform successfully as PACT-related changes are made.	☐	☐	☐	☐	☐

4. Please indicate the number of hours you have spent on each of the following PACT-related activities:

a. Attending local education sessions specifically about PACT.

_____ hours in total

b. Attending regional or national learning collaboratives specifically about PACT.

_____ hours in total

c. Attending the educational activities above with other members of your teamlet. *(If you are not a teamlet member, skip to Q5 on the next page.)*

_____ hours in total

d. Participating in teamlet meetings or huddles.

_____ hours per week (on average)

5. Please answer the following yes/no questions about your exposure to the following changes over the past year. If you answer "yes" to a question, also rate how helpful you found that change for improving care.

	Write Yes, No, or Don't know (If Yes, rate→)	How helpful was/is this change? *(Check one)*		
		Not at all helpful	Somewhat helpful	Very helpful
a. Are you using new measurement tools associated with PACT to help assess your team/teamlet's performance?		☐	☐	☐
b. Do you participate in teamlet huddles?		☐	☐	☐
c. Does your clinic use information systems (e.g., Compass, PCMM) to provide timely data and feedback to staff on PACT activities?		☐	☐	☐
d. Do you receive regular reports or feedback on your performance?		☐	☐	☐
e. Has your clinic implemented new approaches to scheduling?		☐	☐	☐
f. Are you involved in any small tests of change to improve quality of care (e.g., testing out new patient care approaches on a few patients or providers first)?		☐	☐	☐

6. Please indicate how much you agree or disagree with the following statements:

	Strongly disagree	Disagree	Neither disagree nor agree	Agree	Strongly agree
a. PACT-related changes have been helpful for improving patient care.	☐	☐	☐	☐	☐
b. When PACT achieves full development, it will improve patient care.	☐	☐	☐	☐	☐

D. Organizational Context

1. **Please indicate how much you agree or disagree with each of the following statements about your primary care clinic.** *(If you work in more than one clinic, please answer these questions with respect to the clinic you spend the most time working in.)*

	Strongly disagree	Disagree	Neither disagree nor agree	Agree	Strongly agree
a. In this clinic, it is easy to speak up about what is on your mind.	☐	☐	☐	☐	☐
b. In this clinic, people often resist untried approaches.	☐	☐	☐	☐	☐
c. Staff and clinicians are involved in developing plans for improving quality.	☐	☐	☐	☐	☐
d. If you make a mistake in this clinic, it is often held against you.	☐	☐	☐	☐	☐
e. People in this clinic are usually comfortable talking about problems.	☐	☐	☐	☐	☐
f. Clinic leadership discourages nursing staff from taking initiative.	☐	☐	☐	☐	☐
g. People in this clinic are eager to share information about problems and disagreements.	☐	☐	☐	☐	☐
h. In this clinic, when I have a problem that involves a co-worker from a different clinical or administrative discipline, I can access help to resolve the problem.	☐	☐	☐	☐	☐
i. In this clinic, people value new ideas.	☐	☐	☐	☐	☐
j. Unless an idea has been around for a long time, no one in this clinic wants to hear it.	☐	☐	☐	☐	☐
k. When there is a conflict in this clinic, we usually talk it out and resolve the problem successfully.	☐	☐	☐	☐	☐
l. This clinic encourages staff and clinicians' input for making changes and improvements.	☐	☐	☐	☐	☐
m. This clinic defines success as teamwork and concern for people.	☐	☐	☐	☐	☐
n. All of the staff and clinicians participate in important decisions about clinical operations (e.g., workflow).	☐	☐	☐	☐	☐

Please indicate how much you agree or disagree with each of the following statements about your primary care clinic. *(Question 1 continued.)*

	Strongly disagree	Disagree	Neither disagree nor agree	Agree	Strongly agree
o. In this clinic, co-workers from different clinical or administrative backgrounds frequently interact to solve quality of care problems.	☐	☐	☐	☐	☐
p. Our staff and clinicians have constructive work relationships.	☐	☐	☐	☐	☐
q. There is often tension between people in this clinic.	☐	☐	☐	☐	☐
r. The staff and clinicians in this clinic operate as real teams.	☐	☐	☐	☐	☐

2. Please indicate how much you agree or disagree with each of the following statements about your primary care clinic leadership.

Primary care clinic leadership:	Strongly disagree	Disagree	Neither disagree nor agree	Agree	Strongly agree
a. Provides measurable objectives for implementing the strategy and vision within our clinic.	☐	☐	☐	☐	☐
b. Recognizes and rewards progress in implementing change with our clinic.	☐	☐	☐	☐	☐
c. Believes that the current clinic patterns can be improved.	☐	☐	☐	☐	☐
d. Encourages and supports changes in clinic patterns to improve patient care.	☐	☐	☐	☐	☐
e. Is willing to try new clinical protocols.	☐	☐	☐	☐	☐
f. Works cooperatively with senior leadership/clinical management to make appropriate changes.	☐	☐	☐	☐	☐
g. Understands the difficulties and challenges related to the implementation of PACT.	☐	☐	☐	☐	☐
h. Uses measures or evidence from the literature, rather than personal opinions alone, to shape changes in the clinic.	☐	☐	☐	☐	☐

E. Patient-Centered Care

The Institute of Medicine describes "patient-centered care" as health care that respects and honors patients' individual wants, needs, and preferences, and that assures that individual patients' values guide all decisions.

1. Please indicate how well each of the following statements describes your primary care clinic.

Our primary care clinic:	Not at all	Slightly	Moderately	Very	Extremely	Don't know
a. Is patient-centered *(see description above)*	☐	☐	☐	☐	☐	☐
b. Minimizes the waste of resources	☐	☐	☐	☐	☐	☐
c. Makes use of patient feedback (e.g., from patient surveys) for improvement	☐	☐	☐	☐	☐	☐
d. Makes use of clinical performance measure results for improvement	☐	☐	☐	☐	☐	☐
e. Integrates mental health, primary, and substance abuse care	☐	☐	☐	☐	☐	☐
f. Facilitates coordinated care	☐	☐	☐	☐	☐	☐
g. Provides continuity of care	☐	☐	☐	☐	☐	☐

F. Personal Characteristics

1. **What is your age?**

☐☐

Years

2. **What is your gender?**

(Circle one)

Male 1
Female.. 2

3. **Are you Spanish/Hispanic/Latino?**

(Circle one)

No, not Spanish/Hispanic/Latino 1
Yes, Mexican, Mexican-American, Chicano, Puerto Rican, Cuban or
Other Spanish/Hispanic/Latino... 2

4. **What is your race?**

(You may circle more than one)

White 1
Black or African-American.. 2
American Indian or Alaska Native 3
Asian (e.g. Asian Indian, Chinese, Filipino, Japanese,
Korean, Vietnamese) 4
Native Hawaiian or other Pacific Islander (e.g. Samoan,
Guamanian or Chamorro).. 5
Other 6
↳ (*Please specify*: ______________________________)

5. **Have you completed any other survey(s) about health care teams within the past year?**

(Check one) ☐ No ☐ Yes

THANK YOU FOR PARTICIPATING IN THIS STUDY.

Comments (optional):
